# Giant Broad Ligament Leiomyoma: A Diagnostic and Surgical Challenge

**DOI:** 10.7759/cureus.72890

**Published:** 2024-11-02

**Authors:** Sanghamitra Jena, Neetesh K Sinha, Anil Prasad, Krishna Gopal, Jasmine Mallik

**Affiliations:** 1 Surgical Oncology, Tata Main Hospital, Jamshedpur, IND; 2 Pathology, Tata Main Hospital, Jamshedpur, IND

**Keywords:** broad ligament, giant, intra-operative complications, leiomyoma, surgical challenge

## Abstract

Broad ligament leiomyomas are rare tumors present outside the uterine body. The incidence of giant broad ligament leiomyomas (weighing over 3 kg) remains relatively rare. In this report, we present the case of a peri-menopausal woman who came in with a large abdominal mass. She was provisionally diagnosed with a right ovarian mass and planned for an exploratory laparotomy. During the procedure, a large tumor originating from the right broad ligament was seen. The tumor was occupying almost the whole of the abdominal cavity, and we faced lots of challenges to resect it without damaging the ureter and the bowel loops.

This case report highlights the difficulty of diagnosing a giant broad ligament fibroid pre-operatively. It also emphasizes the intra-operative challenges faced during the management of this extremely rare condition.

## Introduction

The most common benign tumors of the uterus are leiomyomas [1]. They comprise 20-30% of all sonologically diagnosed uterine tumors [2]. They are mostly located inside the uterine cavity but can also be present in extra-uterine sites like the cervix or the broad ligament [3]. Broad ligament leiomyomas account for only 6-10% of all uterine leiomyomas [4], and those measuring >20 cm or weighing >3 kg are termed giant leiomyomas. Only six cases of giant broad ligament leiomyomas have been reported in the English literature globally till date [5-8]. In this study, we present a broad ligament leiomyoma weighing 5 kg and measuring 28 cm in diameter.

## Case presentation

A 58-year-old woman presented to the Department of Surgical Oncology with complaints of abdominal swelling that had been progressively increasing in size over the past six months. She had normal bowel and bladder habits and no history of any menstrual irregularities. She had five live births, all delivered vaginally, and the last childbirth was 30 years back.

On examination, the lady was of average body build. A large abdominal mass was palpated, extending from the pelvis up to the xiphisternum. The cervix appeared normal and bilateral fornices were free.

**Figure 1:**
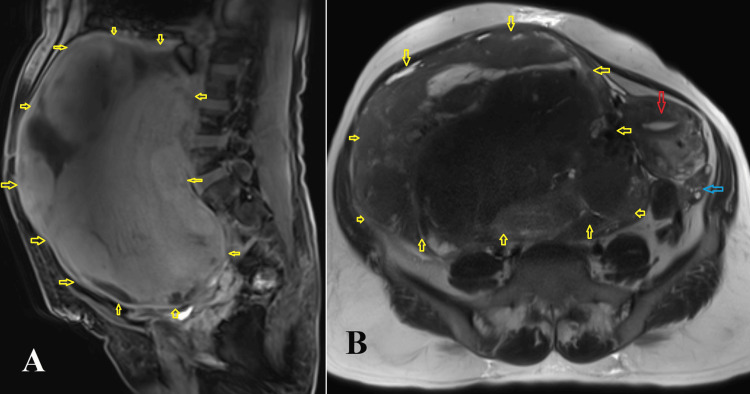
MRI of the abdomen. (A) The sagittal T1 post-contrast scan shows a well-defined mass lesion involving the entire peritoneal cavity extending from the dome of the diaphragm above to the pelvis below. It shows heterogenous enhancement on post-contrast scan. (B) Axial T2-weighted scan shows a large T2 hetero-intense well-defined mass lesion in the right adnexa which is displacing the uterus towards the left side with a well-defined fat plane. Anteriorly, it is abutting the anterior abdominal wall. Posteriorly, it is abutting the lumbosacral spine The yellow arrow marks the tumor, the red arrow marks the uterus, and the blue arrow marks the left ovary. MRI: magnetic resonance imaging

The blood parameters including the tumor markers (CA-125, beta-human chorionic gonadotropin (HCG), lactate dehydrogenase (LDH), and alpha-fetoprotein) were within normal range. Magnetic resonance imaging (MRI) of the abdomen showed a 24.5 × 19.7 cm large well-defined heterogenous soft tissue lesion in the abdominopelvic cavity probably arising from the right adnexa (Figure 1). It showed heterogenous signal intensity on T1- and T2-weighted images and post-contrast enhancement with internal non-enhancing necrotic areas. There was a uterine fibroid in the fundal region measuring 5 × 4.7 cm. The left ovary was normal and displaced to the left iliac fossa. The right ovary was not visualized separately. The chest imaging was normal.

**Figure 2:**
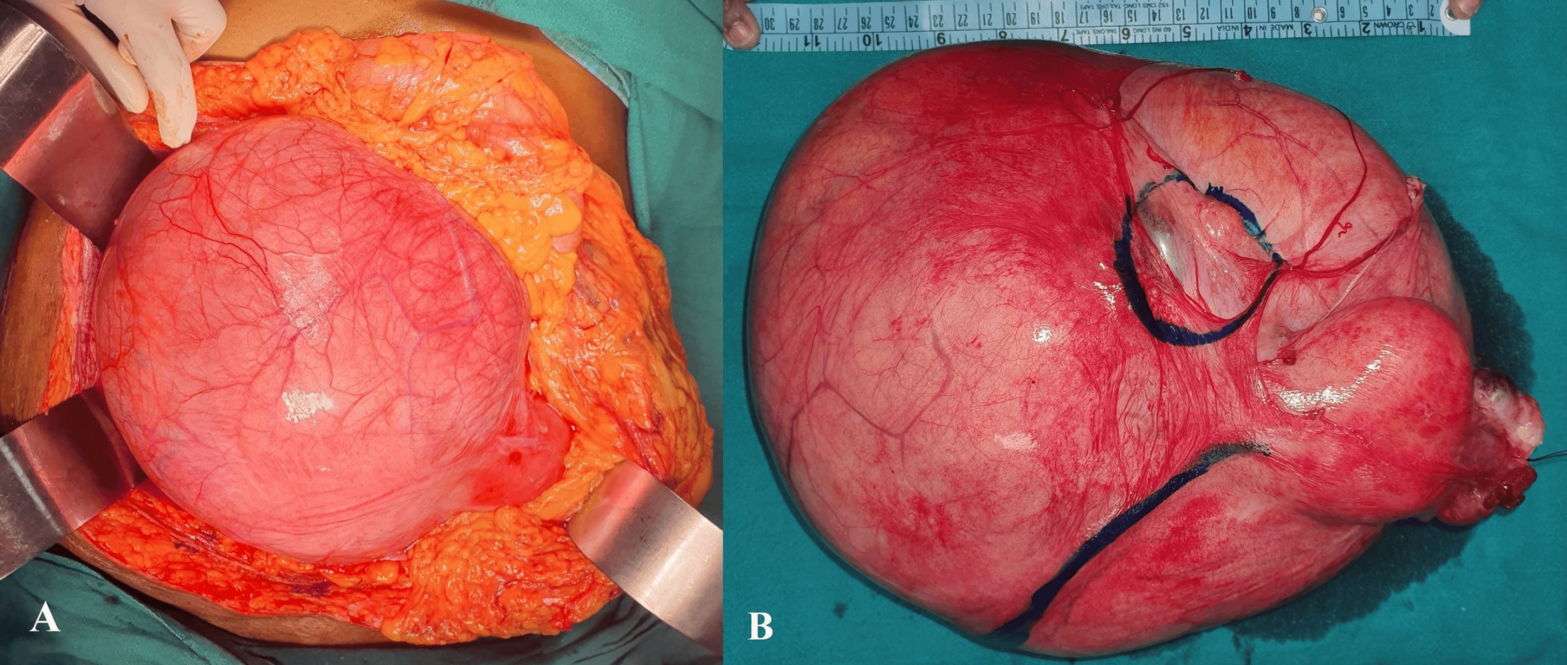
(A) Intra-operative picture showing a large abdominopelvic mass and pushing the bowel loops superiorly. (B) Specimen of total abdominal hysterectomy and bilateral salpingo-oophorectomy. The left ovary is marked with a suture and the right ovary and round ligament are marked with ink

Based on the clinic-radiologic suspicion of the right ovarian mass, the patient was planned for laparotomy with the consent for total abdominal hysterectomy and bilateral salpingo-oophorectomy. Anticipating chances of ureteral injury because of the large abdominal mass, bilateral ureteric stents were placed pre-operatively. On exploration with a midline incision, a huge, encapsulated mass was seen covering almost the entire peritoneal cavity (Figure 2A). It was displacing the bowel loops upwards towards the hypochondrium. The overlying vessels were dilated. The mass was slowly dissected all around with the help of Harmonic and bipolar cautery. The ureteric stents were palpated intermittently to delineate the course of the ureter. As the dissection proceeded, both ovaries were visualized separately from the tumor. The tumor was then diagnosed to be arising from the right broad ligament. The tumor was excised in total along with hysterectomy and bilateral salpingo-oophorectomy (Figure 2B). Myomectomy was not done separately in this case. Judicious use of vessel sealing devices and suture ligation was done while dividing the infundibulopelvic and round ligaments. Hemostasis was achieved and an abdominal drain was placed in the pelvis. The post-operative recovery was uneventful, and the patient was discharged on the fifth post-operative day.

On histopathological examination, the mass measured 28 × 25 × 17 cm. It was mostly solid with focal mucoid areas. A fibroid was seen in the left posterior body of the uterus, measuring 1.5 cm in diameter.

**Figure 3:**
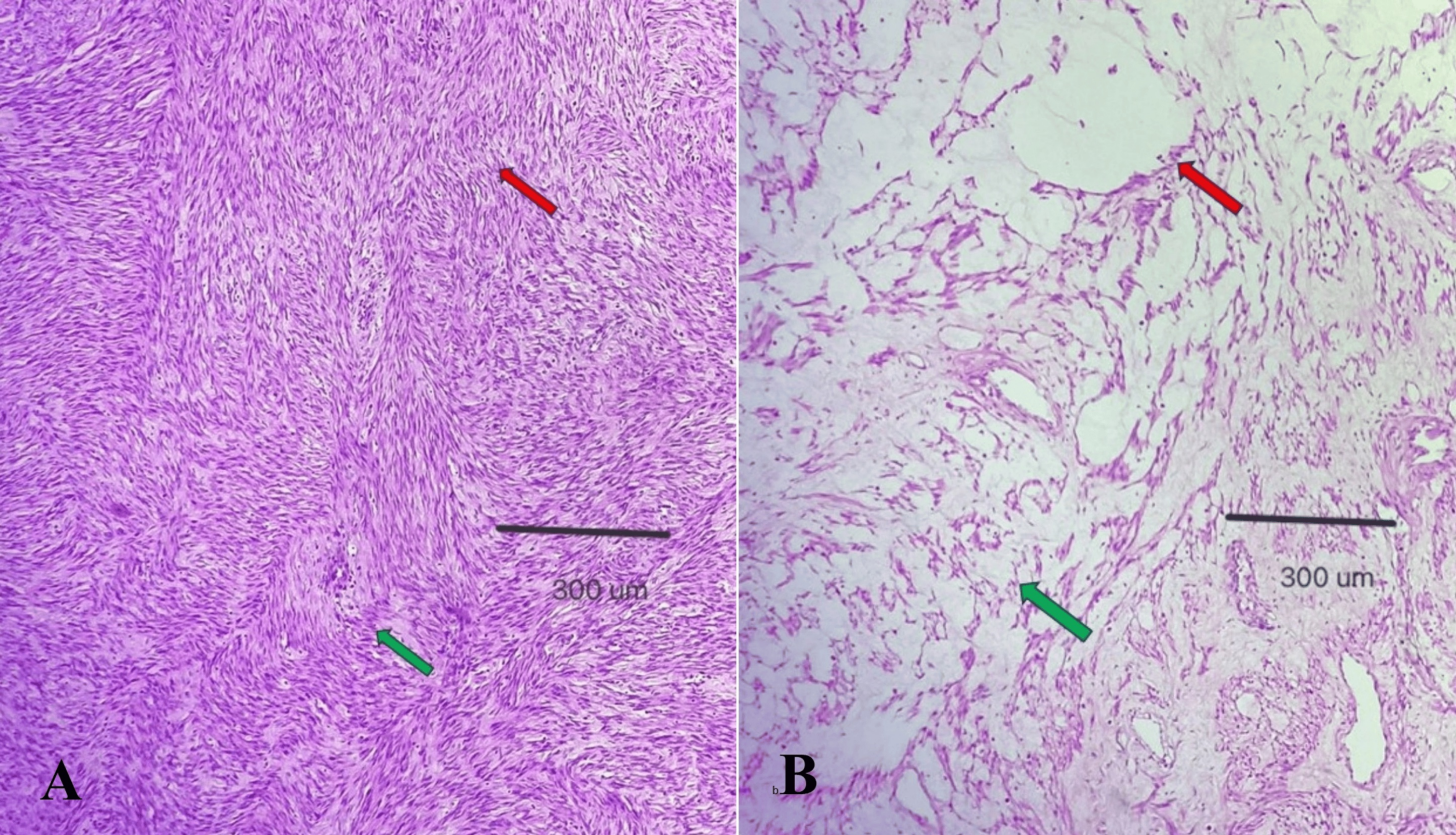
Histopathological images of the hysterectomy specimen (A) Low-power photomicrograph demonstrating benign spindle cells arranged in interlacing fascicles (green arrow), whorled with groups of cells having their long axis perpendicular to one another (pink arrow): 100× H&E, scale bar 300 µm. (B) Low-power photomicrograph demonstrating leiomyoma with fatty degeneration (green arrow) and cystic changes (pink arrow): 100× H&E, scale bar 300 µm. H&E: hematoxylin and eosin

Microscopically, the tumor showed an intersecting bundle of spindle cells with benign elongated nuclei and eosinophilic cytoplasm. At places mucoid and hyaline degeneration with collagenized areas were seen. The surrounding stroma was fibrous and fibrocollagenous (Figure 3). The mitotic activity was low, and areas of necrosis were not seen. So, the final diagnosis of broad ligament leiomyoma with degenerative changes was made.

## Discussion

Broad ligament leiomyomas are very difficult to diagnose based on their clinical presentations. Most of them are asymptomatic [4]. They may present with features like abdominal pain, distension, constipation, frequent urination, urinary retention, and irregular menstruation when they are enormously large. That is why most of the patients report very late for treatment.

Broad ligament leiomyomas are usually confused with pedunculated subserosal fibroids and solid ovarian malignancies. The differentiation is supported by imaging techniques like transvaginal ultrasound, contrast-enhanced computed tomography, or MRI of the abdomen. In transvaginal ultrasound, the ovaries and uterus are seen separately from the mass [9]. MRI findings of tumor shape, the attachment of the tumor to the uterus, ovary elevation on the side of the tumor, and the separation of the round ligament from the ipsilateral uterine artery may be criteria for differentiating broad ligamental leiomyomas from subserosal fibroids [10].

The diagnosis in most cases is confirmed intra-operatively. Because of the large size and location of the tumor, there is always a high probability of damage to the adjacent intestine, ureter, and urinary bladder. So, pre-operative placement of ureteric stents plays a vital role in the management of such cases [11]. Many surgeons advocate myomectomy prior to hysterectomy to ease the dissection [12]. This gives space for hysterectomy and avoids injury to the neighboring structures. Another common intra-operative complication is bleeding. This occurs due to the dilatation of the vascular plexus in the cardinal ligaments which gets engorged as the tumor grows. The use of Harmonic and bipolar vessel sealing devices (LigaSure, EnSeal) reduces intra-operative blood loss. Wang et al. have also shown that combining two novel laparoscopic ligation techniques can be a safe alternative for reducing intra-operative hemorrhage [13].

## Conclusions

Giant broad ligament leiomyomas are a diagnostic challenge because of their rare presentation and clinic-radiologic resemblance with ovarian neoplasms. This case report emphasizes the importance of considering broad ligament leiomyomas as a potential differential diagnosis when encountering a huge abdominal mass. This is essential because resection of these tumors needs surgical expertise. 
